# Maternal complications and cesarean section without indication: systematic review and meta-analysis

**DOI:** 10.11606/S1518-8787.2017051000389

**Published:** 2017-11-13

**Authors:** Keila Cristina Mascarello, Bernardo Lessa Horta, Mariângela Freitas Silveira

**Affiliations:** I Universidade Federal de Pelotas. Centro de Pesquisas Epidemiológicas. Programa de Pós-Graduação em Epidemiologia. Pelotas, RS, Brasil; II Universidade Federal do Espírito Santo. Departamento de Ciências da Saúde. São Mateus, ES, Brasil; III Universidade Federal de Pelotas. Faculdade de Medicina. Departamento Materno Infantil. Pelotas, RS, Brasil

**Keywords:** Puerperal Disorders, epidemiology, Maternal Mortality, Risk Factors, Cesarean Section, contraindications, Natural Childbirth, Meta-Analysis, Transtornos Puerperais, epidemiologia, Mortalidade Materna, Fatores de Risco, Cesárea, contraindicações, Parto Normal, Metanálise

## Abstract

**OBJECTIVE:**

The objective of this study was to determine the risks of severe acute maternal complications associated with cesarean section without medical indication.

**METHODS:**

A systematic review was carried out with meta-analysis. The literature search was performed systematically, in multiple stages, in the PubMed, Lilacs, and Web of Science databases using the following descriptors: (postpartum period) and (cesarean section or natural childbirth) and ((morbidity or mortality) or (postpartum hemorrhage) or (puerperal infection) or (surgical infection) or (puerperal disorders)). The protocol of the study was registered at PROSPERO as CRD42016032933. A total of 1,328 articles were found; after selection, eight publications that met the study objective and inclusion criteria were selected, with information on 1,051,543 individuals.

**RESULTS:**

The results obtained in the meta-analyses indicate that women with cesarean section have a higher chance of maternal death (OR = 3.10, 95%CI 1.92–5.00) and postpartum infection (OR = 2.83, 95%CI, 1.585.06), but they have a lower chance of hemorrhage (OR = 0.52, 95%CI 0.48–0.57). For the blood transfusion outcome, the group effect was not associated with the type of delivery (95%CI 0.88–2.81).

**CONCLUSIONS:**

The quality of evidence was considered low for hemorrhage and blood transfusion and moderate for postpartum infection and maternal death. Thus, cesarean sections should be performed with caution and safety, especially when its benefits outweigh the risks of a surgical procedure.

## RESUMO

**OBJETIVO:**

Determinar os riscos de complicações maternas agudas graves associadas ao parto cesárea sem indicação médica.

**MÉTODOS:**

Foi conduzida uma revisão sistemática com meta-análise. A busca na literatura ocorreu de forma sistemática, em múltiplas etapas, nas bases de dados PubMed, Lilacs e Web of Science, utilizando os descritores: (postpartum period) and (cesarean section or natural childbirth) and ((morbidity or mortality) or (postpartum hemorrhage) or (puerperal infection) or (surgical infection) or (puerperal disorders)). O protocolo de estudo foi registrado na PROSPERO sob o número CRD42016032933. Foram encontrados 1.328 artigos, permanecendo, após seleção, oito publicações que atendiam ao objetivo do estudo e critérios de inclusão, com informações de 1.051.543 indivíduos.

**RESULTADOS:**

Os resultados obtidos nas meta-análises indicam que mulheres de parto cesárea tem maior chance de morte materna (OR = 3,10; IC95% 1,92–5,00) e infecção pós-parto (OR = 2,83; IC95% 1,58–5,06), mas possuem menor chance de hemorragia (OR = 0,52; IC95% 0,48–0,57). Para o desfecho transfusão de sangue, o efeito agrupado não foi associado à via de parto (IC95% 0,88–2,81).

**CONCLUSÕES:**

A qualidade da evidência foi considerada baixa para os desfechos hemorragia e transfusão de sangue e moderada para infecção pós-parto e morte materna. Assim, as cesáreas devem ser realizadas com prudência e segurança, principalmente quando seus benefícios superam os riscos de um procedimento cirúrgico.

## INTRODUCTION

The rates of cesarean section have increased significantly in recent decades^6^. In 2008, 6.2 million unnecessary cesarean sections were performed worldwide; China and Brazil represent approximately 50% of all cesarean sections without medical indication^15^.

Brazil has significantly increased the rates of cesarean section in recent decades. Estimates from 1970 indicate that this rate was approximately 15%, rising to 38% in 2001 and to 48.8% in 2008; cesarean sections represented 35% of the deliveries in the Brazilian Unified Health System (SUS) and 80% of the deliveries in the private sector^28^. In 2009, the rate was 50.1%, surpassing, for the first time, the number of vaginal deliveries. This number continues to increase, and cesarean sections represented 55.7% of the births in 2012^21^.

This increase in the number of cesarean sections worldwide is related to the improvement of the access of women to this procedure when needed, but it is also related to the indiscriminate use without medical indication. This has culminated in the recent efforts to reduce these rates, while incorporating the obstetric preferences of women^3^.

Properly performed cesarean sections that follow an accurate medical indication are life-saving procedures. However, on the one hand, the provision of safe and timely cesarean sections remains a major challenge in countries with high maternal mortality, where they are insufficient^25^; on the other hand, their excess in certain regions results in the challenge of minimizing cesarean sections without clinical indication.

Despite the undeniable importance of this procedure, pregnant women and health professionals need to know the maternal risks associated with the different types of deliveries, using the best evidence^17^. Therefore, this review is justified by the need to synthesize knowledge about the frequency of acute maternal complications associated with cesarean section without clinical indication, which will assist in counseling women who examine the advantages and disadvantages of this type of procedure compared to vaginal delivery.

Thus, this study aimed to perform a systematic review and meta-analysis to determine the risks of severe acute maternal complications associated with cesarean section without medical indication compared to vaginal delivery.

## METHODS

To identify the studies that evaluated acute maternal complications associated with the type of delivery, we revised the PubMed, Lilacs, and Web of Science databases in January 2016 to search for articles without date or language restriction. The search strategy to identify the studies included the use of the Medical Subject Heading (MeSH) and the Health Sciences Descriptors (DeCS). The descriptors used as MeSH and DeCS were: (postpartum period) and (cesarean section or natural childbirth) and ((morbidity or mortality) or (postpartum hemorrhage) or (puerperal infection) or (surgical infection) or (puerperal disorders)). As an additional resource, we searched records in the references of the selected articles.

We included studies evaluating acute complications, usually occurring up to 42 days postpartum, related to the type of delivery, including only the cesarean sections reported as with no medical indication or in women of low obstetric risk, without previous complications, or that presented this information separately, compared to vaginal delivery. We exclude articles that did not measure the outcomes of this study (acute maternal complications associated with the type of delivery, including hemorrhage, hysterectomy, blood transfusion, hospitalization in intensive care unit, postpartum infection, hospitalization for more than seven days, obstetric trauma, previously defined), those that measured only neonatal complications associated with the type of delivery and not acute maternal complications, those that measured only postpartum psychiatric disorders, and the records referring to editorials or service protocols.

We defined a protocol to extract the data from the complete texts, with year of publication, country of study, study design, sample size, objectives of the study, inclusion and exclusion criteria, controls used for confounding factors, and main results. The process of selecting the references and extracting the results was carried out by two independent researchers and the disagreements were discussed in person.

The study protocol was submitted to the International Prospective Register of Systematic Reviews (PROSPERO), and it was approved as CRD42016032933. In the preparation of this article, we followed the recommendations of Preferred Reporting Items for Systematic Reviews and Meta-Analyses (PRISMA)^22^.

The quality of the selected articles was evaluated according to the adapted instrument of Downs and Black^13^. The original version consists of twenty-seven items, but for this study the questions related to experimental studies were excluded; thus, we used seventeen items: 1) Was the hypothesis/objective of the study clearly defined?; 2) Are the main measured outcomes clearly described in the introduction or methods?; 3) Are the characteristics of the individuals clearly described?; 4) Is the distribution of the main confounding factors on the subject to be compared clearly described?; 5) Are the main findings of the study described?; 6) Does the study provide estimates of random variability of the data for the main outcomes (measures of variability)?; 7) Are the characteristics of the patients who were losses, refusals, and monitoring losses described?; 8) Are the p values described “accurately” rather than, for example, p < 0.05, except for p < 0.001?; 9) Are the subjects invited to participate in the research representative of the population from which they were recruited?; 10) If any of the results of the study was based on data dredging, was it clearly done?; 11) Were the statistical tests suitable to evaluate the main outcomes?; 12) Was the main outcome measured using accurate criteria/equipment (valid and replicable)?; 13) Were the study participants recruited in the same time period?; 14) Were the groups to be compared obtained from the same population?; 15) Were the confounding adjustments appropriate in the analysis from where the main findings were obtained?; 16) Were the monitoring losses taken into account?; 17) Does the study have enough power to detect an important clinical effect when the value of the probability for the difference from chance is less than 5%? All the question addressed receive one point for “yes” and zero for “no”, except question four, with zero for “no”, one for “partially”, and two for “yes”, resulting in a score from zero to eighteen points.

All selection steps, except full reading, were performed in the EndNote program (Thomson Reuters http://www.endnote.com/) after importing the search results from the databases into a library in the program.

For the outcomes of blood transfusion, death, postpartum infection, and hemorrhage, a meta-analysis was possible, as they had two or more comparable studies. A new review of the articles was done to ensure that the data of each individual or population were inserted only once in the quantitative analysis. The combined odds ratio was calculated using the fixed model and, if heterogeneity between studies was high (if the p value of the Q test of heterogeneity was < 0.05 or I^2^ > 50%), the randomized model was used to combine the studies.

In order to analyze the quality of evidence for each outcome included in the meta-analysis, we used the Grading of Recommendations Assessment, Development, and Evaluation (GRADE), defined from the outline of the studies included and the results found. For observational studies, the quality of evidence begins as low, and based on criteria such as methodological limitations, inconsistency of results, indirect evidence, inaccuracy, and publication bias, the level of evidence can be reduced or increased^16^. The quality of evidence obtained using the GRADE system allows the analysis of the aggregate results, considering the design and results of the studies included and the estimation of the group effect obtained by the meta-analysis^16^.

## RESULTS

The search strategy retrieved 1,007 titles in PubMed, six in Lilacs, and 315 in the Web of Science, amounting to 1,328 publications. We excluded 308 duplicates, amounting to 1,020 titles. After reading the titles, 69 abstracts were selected for analysis. The complete flowchart of the selection of articles is shown in the Figure. We also carried out the review of the references of the selected articles, in order to locate articles not captured by the search in the databases, allowing the inclusion of nine other publications in the selection process. At the end of the process, eight articles were included in the review, providing information on 1,051,543 individuals.

**Figure:**
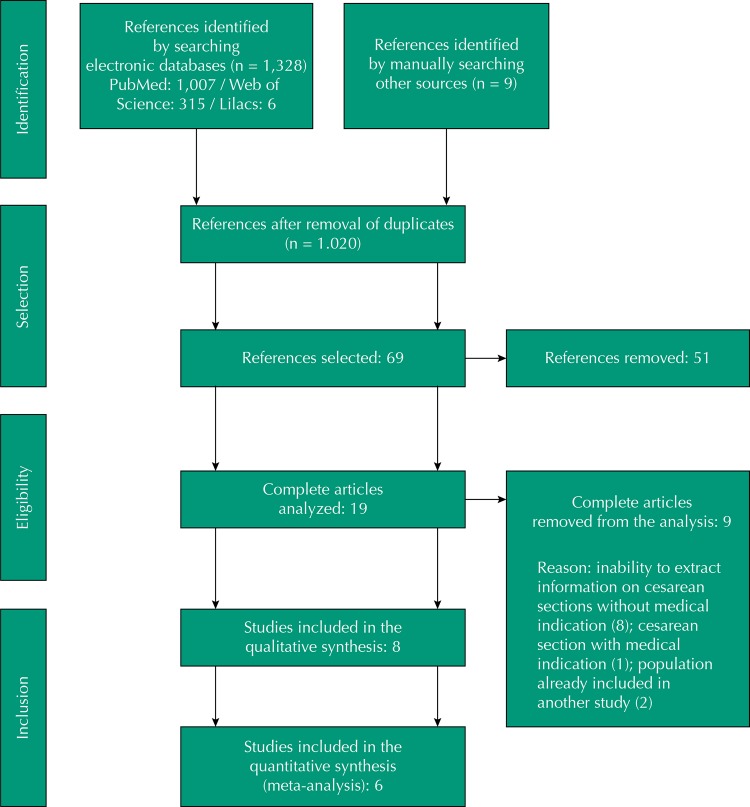
Selection of articles included in the systematic review and meta-analysis.

Most studies (six) were conducted in high-income countries; however, a multicenter study of 24 countries, including low- and middle-income countries^27^, and another study in India^18^ contributed with approximately 25% of the population included in this review. Except for two studies^1^ ^,^ ^19^, the other ones are recent, published in the last 10 years. All of them have evaluated acute obstetric complications as outcome.

Most of the studies were of the longitudinal type, with retrospective secondary data (six) from large health systems databases or medical record review. One study was a case-control and another one was cross-sectional. No prospective study was found.

The exposure of interest (type of delivery) has been examined in different ways among the studies. Most (six) have evaluated exposure dichotomously, such as cesarean section or vaginal delivery^1^ ^,^ ^2^ ^,^ ^11^ ^,^ ^12^ ^,^ ^14^ ^,^ ^19^. The other studies (two) have evaluated exposure in different categories of cesarean section: cesarean with or without indication, before (antepartum cesarean) or after labor began (intrapartum cesarean), or primary or repeat cesarean^18^ ^,^ ^27^.

Similarly, the outcomes were different among studies, although all of them have evaluated immediate puerperal complications. Most of the studies have evaluated the presence of severe puerperal complications, such as severe hemorrhage and blood transfusion, hospitalization in an intensive care unit, hysterectomy, infection, hospitalization for more than seven days, and death.

The Box summarizes the methodological characteristics and main results of the selected articles, arranged in chronological order according to the date of publication.

**Box:** Characteristics of the studies, main results, and Downs and Black scores.

Author, year of publication, and country of research	Type of study, sample size, year of research	Results	Downs and Black score
Allen et al.^1^ (2003), Canada	Retrospective cohort, 18,435, 1988–2001	There was no significant difference in the relative risk of maternal complications in women with cesarean section without labor compared to spontaneous vaginal delivery for blood transfusion, hematoma drainage, postpartum hemorrhage, and intraoperative trauma; women with cesarean section without labor had a higher risk of puerperal infection (RR = 5.4, 95%CI 2.4–11.8) and surgical wound infection (RR = 3.5, 95%CI 1.8–6.7).	11
Koroukian^19^ (2004), United States	Retrospective cohort, 168,736, 1991–1996	Women submitted to elective cesarean section in the absence of risk factors and complications had a higher risk of puerperal infection (RR = 3.75, 95%CI 3.12–4.51), thromboembolic events (RR = 3.45, 95%CI 1.70–7.00), anesthetic complications (RR = 4.43, 95%CI 2.68–7.34), and complications of surgical wound (RR = 12.50, 95%CI 10.00–15.63) and they presented a lower risk of postpartum hemorrhage (RR = 0.60, 95%CI 0.48–0.76) and obstetric trauma (RR = 0.16, 95%CI 0.16–0.20). Blood transfusion was not associated with the type of delivery.	14
Allen et al.^2^ (2006), Canada	Retrospective cohort, 5,779, 1988–2003	There was no statistically significant difference in postpartum infection rates and operative wound, puerperal infection, hematoma drainage, and intraoperative trauma among women with cesarean section and induced vaginal delivery. The women of the cesarean group had a lower chance of postpartum hemorrhage 0.61 (95%CI 0.42–0.88).	15
Deneux-Tharaux et al.^12^ (2006), France	Case-control, 10,309 (65 cases), 1996–2000	Cases of maternal death were more likely in cesarean section than controls. Women with cesarean section presented 3.64 (95%CI 2.15–6.19) times more chance of death than women with vaginal deliveries, with a 3.11 (95%CI 1.58–6.10) chance for antepartum cesarean section and 4.35 (95%CI 2.23–8.45) chance for intrapartum cesarean section.	16
Declercq et al.^11^ (2007), United States	Retrospective cohort, 244,088, 1998–2003	Women of primary cesarean section without labor had a 2.25 times greater chance of rehospitalization in the first 30 days after delivery (95%CI 1.74–2.90) compared to women with vaginal delivery.	12
Souza et al.^27^ (2010), 24 countries	Cross-sectional study, 286,565 2004–2008	Women who underwent cesarean section after labor, without medical indication, did not present a higher risk of death than women with spontaneous vaginal delivery (OR = 3.21, 95%CI 0.78–13.2), but they had a greater chance of admission to an intensive care unit (ICU) (OR = 58.85, 95%CI 41.46–83.52), blood transfusion (OR = 2.24, 95%CI 2.24–6.1), hysterectomy (OR = 13.53, 95%CI 4.79–38.2), and other serious maternal outcomes (OR = 14.29, 95%CI 10.91–18.72). Cesarean section before labor and without indication led to a greater chance of admission to ICU (OR = 30.75, 95%CI 18.12–52.17) and other serious maternal outcomes (OR = 5.93, 95%CI 3.88–9.05). Regardless of medical indication, cesarean section was not protective for any of the outcomes analyzed.	16
Farchi et al.^14^ (2010), Italy	Retrospective cohort, 273,789, 2001–2007	Women with low-risk pregnancies submitted to cesarean section had a higher chance of hysterectomy (OR = 1.30, 95%CI 1.01–1.66), obstetric shock (OR = 2.15, 95%CI 1.14–4.07), and complications of anesthesia (OR = 2.18, 95%CI 1.02–4.65). Cesarean section was a protection for uterine rupture among multiparous women (OR = 0.29, 95%CI 0.15–0.58). There was no significant difference for postpartum infection (OR = 1.46, 95%CI 0.89–2.40).	14
Kamilya et al.^18^ (2010), India	Retrospective cohort, 43,842, 2003–2006	The women with cesarean section, in the absence of complications and comorbidities, presented a 3.01 times higher death rate than women with vaginal delivery (95%CI 1.66–5.46). When cesarean section was intrapartum, this chance was 4.86 (95%CI 2.47–9.56) and, for cesarean section before labor, this chance was not significantly higher (OR = 1.73, 95%CI 0.80–3.71).	11

### Postpartum Infection

The presence of postpartum infection has been evaluated in four studies^1^ ^,^ ^2^ ^,^ ^14^ ^,^ ^19^. Among them, one has found no association between the type of delivery and the presence of infection (OR = 1.46, 95%CI 0.89–2.40)^14^, and the others have found a higher risk of puerperal infection (RR = 3.75, 95%CI 3.12–4.51) and surgical wound complications (RR = 12.50, 95%CI 10.00–15.63) among women undergoing cesarean section compared to vaginal delivery^19^; another study has shown that, in cesarean sections before labor, women presented a higher risk of puerperal infection (RR = 5.4, 95%CI 2.4–11.8) and surgical wound infection (RR = 3.5, 95%CI 1.8–6.7)^1^.

### Hemorrhage and Blood Transfusion

Six studies have evaluated the presence of postpartum hemorrhage and its complications, such as hysterectomy and blood transfusion, and they have found controversial results. Two studies have found a lower risk of postpartum hemorrhage among women with cesarean section, with similar estimates (RR = 0.60; 95%CI 0.48–0.76^11^ and RR = 0.61, 95%CI 0.42–0.88^2^); however, another study has found no association between type of delivery and hemorrhage and type of delivery and blood transfusion^1^.

The chance of blood transfusion (as a possible consequence of severe hemorrhage) was higher among women undergoing cesarean section after labor (OR = 2.24, 95%CI 2.24–6.1)^27^. Increased chance of transfusion was not found among women with antepartum cesarean section, who also had no greater chance of hysterectomy.

One study has found a higher chance of hysterectomy in women with intrapartum cesarean section (OR = 13.53, 95%CI 4.79–38.2)^27^ and, to a lesser extent, in cesarean sections in general (OR = 1.30, 95%CI 1.01–1.66)^14^.

### Hospitalization in Intensive Care Unit

The need for hospitalization in intensive care unit (ICU) as a predictor of severe complications has been evaluated in a large study by the World Health Organization (WHO) linking data from 24 countries. It has shown that women undergoing cesarean section were more likely to be admitted to the ICU, be it intrapartum (OR = 58.85, 95%CI 41.46–83.52) or antepartum cesarean section (OR = 30.75, 95%CI 18.12–52.17)^27^.

Women of primary cesarean section without labor also had a 2.25 times greater chance of rehospitalization in the first 30 days after delivery (95%CI 1.74–2.90) compared to women with vaginal delivery^11^.

### Obstetric Trauma

Only one study has evaluated the presence of obstetric trauma, including perineal and vaginal laceration, other pelvic organ damage and damage to pelvic joints and ligaments, showing that women with vaginal deliveries were more likely to experience this complication when compared to women undergoing cesarean section (RR = 0.09, 95%CI 0.07–0.11)^19^.

### Maternal death

Among the studies that have evaluated death^12^ ^,^ ^18^ ^,^ ^27^, one of them^27^ has found no relation between the type of delivery and the chance of death and the others have identified a greater chance of death among women undergoing cesarean section.

In one study, cases of maternal death were more likely in those who underwent surgery than controls (OR = 3.64, 95%CI 2.15–6.19), which was 3.11 times greater (95%CI 1.58–6.10) for antepartum and 4.35 times greater (95%CI 2.23–8.45) for intrapartum cesarean section^12^. A similar result was found in another study in which women with cesarean section, in the absence of complications and comorbidities, presented a 3.01 times higher death rate than women with vaginal delivery (95%CI 1.66–5.46). When the cesarean section was intrapartum, this chance was 4.86 times higher (95%CI 2.47–9.56); however, for antepartum cesarean section, there was no association (OR = 1.73, 95%CI 0.80–3.71)^18^.

Table 1 describes the results of the meta-analyses for the outcomes evaluated. The group effect shows that women with cesarean section have a higher chance of maternal death (OR = 3.10; 95%CI 1.92–5.00) and postpartum infection (OR = 2.83, 95%CI 1.58–5.06), but they have a lower chance of hemorrhage (OR = 0.52, 95%CI 0.48–0.57). For the blood transfusion outcome, the group effect was not associated with the type of delivery (95%CI 0.88–2.81).

**Table 1:** Meta-analysis of studies on acute maternal complications associated with cesarean section without clinical indication.

Outcome	Effect estimate	95%CI	Weight (%)	p^b^
Postpartum infection				
Allen et al.^1^ (2003)	2.2	1.08–4.45	21.71	
Koroukian^19^ (2004)	4.07	3.71–4.46	31.69	< 0.001
Allen et al.^2^ (2006)	4.87	2.28–10.37	20.69	
Farchi et al.^14^ (2010)	1.46	0.88–2.39	25.9	
Group effect	2.83	1.58–5.06	100	
Hemorrhage				
Allen et al.^1^ (2003)	0.60	0.40–0.90	5.17	
Koroukian^19^ (2004)	0.51	0.462–0.56	87.94	0.146
Allen et al.^2^ (2006)	0.72	0.50–1.02	6.89	
Group effect	0.52	0.48–0.57	100	
Maternal death				
Souza et al.^27^ (2010)				
Antepartum cesarean	not estimated^a^			
Intrapartum cesarean	3.21	0.78–13.20	11.41	
Kamilya et al.^18^ (2010)				0.141
Antepartum cesarean	1.73	0.80–3.72	38.77	
Intrapartum cesarean	4.86	2.47–9.56	49.82	
Group effect	3.10	1.92–5.00	100	
Blood transfusion				
Allen et al.^1^ (2003)	0.70	0.19–2.57	10.53	
Koroukian^19^ (2004)	1.86	1.37–2.51	21.13	< 0.001
Allen et al.^2^ (2006)	1.85	0.51–6.68	10.68	
Souza et al.^27^ (2010)				
Antepartum cesarean	1.79	0.91–3.52	17.18	
Intrapartum cesarean	3.70	2.24–6.10	19.20	
Farchi et al.^14^ (2010)	0.77	0.58–1.02	21.28	
Group effect	1.57	0.88–2.81	100	

^a^ No cases of maternal death in the group.

^b^ Chi-square of heterogeneity.

### Quality of Included Studies and Quality of Evidence

The studies included in this review are of good methodological quality, with Downs and Black scores varying between 11 and 16 points. The inclusion of only studies in which cesarean sections were performed without medical indication and with women of low obstetric risk reduced the number of potential confounding factors and it allowed for risks associated with the procedure to be from complications prior to the cesarean.

All studies carried out control for potential confounding factors, but in two of them^14^ ^,^ ^18^ this adjustment was considered insufficient as they controlled only maternal age and parity^18^ and age, education level, and country of birth^14^. Adequate adjustment should consider at least the variables of maternal age, race, education level, parity, and diseases in the current gestation, either in the study design or during the analyses.

The main limitation found in the included studies is the difficulty in adequately assessing complications and outcomes. Most of the studies were carried out using secondary data from large databases and they depended on the evaluation of the professional who assisted the woman; thus, they did not have accurate, valid, and replicable criteria.

Table 2 shows the quality of evidence according to the GRADE system^16^. For the postpartum infection and maternal death outcomes, the quality of the evidence is moderate; that is, there is moderate confidence in the estimated effect that shows greater chance in women who underwent cesarean section. For the hemorrhage outcome, the quality of evidence is low and, for blood transfusion, the quality is very low.

**Table 2:** Summary of results for the quality of evidence according to the GRADE system.

Outcome	Group effect (95%CI)	Participants (number of studies)	Quality of evidence (GRADE)^16^
Postpartum infection	2.83 (1.58–5.06)	466,739 (4)	Moderate^a^
Hemorrhage	0.52 (0.48–0.57)	236,793 (3)	Low
Maternal death	3.10 (1.92–5.00)	257,640 (2)	Moderate^a^
Blood transfusion	1.57 (0.88–2.81)	682,271 (5)	Very low^b^

GRADE: Grading of Recommendations Assessment, Development, and Evaluation

^a^ Low level (observational studies) + consistent findings (1 level).

^b^ Low level (observational studies) - inconsistent findings (1 level).

## DISCUSSION

This systematic review identified eight studies that evaluated early puerperal complications and type of delivery. The currently available evidence is still controversial for the outcomes of blood transfusion, which has very low quality of evidence, and hemorrhage, with low quality of evidence, since the findings were not consistent among each other. For the postpartum death and infection outcomes, the results were similar in the different studies.

The presence of postpartum infection, regardless of the site of infection, not specified in the studies, or infection of the surgical wound, was higher in cesarean sections, as well as the need for hospitalization in the ICU. The risk of hemorrhage, hysterectomy, and blood transfusion seems to be greater only in intrapartum cesarean sections; however, one study has found an increased risk of hemorrhage among women with vaginal delivery. The risk of death is also inconclusive.

Regarding hysterectomy, we should take into account the possibility of this procedure being programmed, not because of a complication of delivery, especially in large studies with secondary data and cross-referencing, without direct access to the patient, even if in a small number of cases. Another systematic review aimed at determining the relationship between cesarean section and emergency hysterectomy has found that cesarean section is a risk factor for the procedure and the risk increases with each additional cesarean section^10^. A meta-analysis that has evaluated the presence of early complications has shown that the planned cesarean section was associated with a lower risk of urinary incontinence and blood transfusion and a higher risk of hemorrhage.

The risk of obstetric trauma was also higher among women with vaginal delivery, which may be reflected late on the health and quality of life of these women, increasing, for example, the risk of future urinary incontinence^9^. A recent systematic review has shown that genital prolapse and urinary incontinence were less prevalent in women who have only cesarean sections^26^.

The differences between studies need to be considered, especially for the maternal death and hemorrhage outcomes. Among the studies that have evaluated maternal death, no association was found in those that have controlled for a greater number of confounding factors and that had larger samples. It is worth mentioning that the quantitative analysis of the studies found a higher risk of death among women with cesarean section.

Other studies that evaluate the risk of hemorrhage and the type of delivery should be conducted to clarify this point. Women with cesarean section had a lower risk of hemorrhage than women with vaginal delivery in the meta-analysis; however, other studies have suggested that women with cesarean section are at greater risk for blood transfusion and hysterectomy, suggesting that hemorrhage is more severe in these women^14^ ^,^ ^27^. These differences may be due to the difficulty in measuring the amount of blood lost or even an underestimation of blood loss during cesarean section, or, on the other hand, it may be due to an increase in blood loss related to episiotomy or perineal or vaginal trauma in the vaginal delivery.

Risks to the fetus and newborns should also be considered during the process of choosing the type of delivery in the absence of a medical indication for cesarean section. A review comparing cesarean section without medical indication and vaginal delivery has shown that cesarean section increases the risk of respiratory complications in the newborn^7^. Increases in cesarean rates have also been associated with higher rates of fetal mortality and a higher number of newborns admitted to a neonatal ICU for seven days or more, even after controlling for prematurity^29^.

Since 1985, the WHO has warned that there is no justification for cesarean section rates above 10%–15% of all deliveries^31^, although more studies are needed to confirm or refute this recommendation. A rediscussion on the subject held in 2014 found similar results; however, the main recommendation now is to provide appropriate cesarean sections to women who would really need and benefit from the surgical delivery, rather than following a specific rate^32^.

Several ecological studies have been carried out in an attempt to find an association between the percentage of cesarean sections and maternal morbidity and mortality. Among them, a recent survey with 194 countries, members of the WHO, suggests that the recommended 10% to 15% rate may be very low, as it found maternal and neonatal mortality rates inversely proportional to cesarean rates up to 19.1 per 100 live births (95%CI 16.3–21.9) and 19.4 per 100 live births (95%CI 18.6–20.3), respectively^23^.

Some countries have low maternal and neonatal mortality rates and, at the same time, low cesarean rates. France has a maternal mortality rate of 17 per 100,000 live births and a percentage of cesarean sections of 18.8%. Japan has a maternal mortality rate of 10 per 100,000 live births and a percentage of cesarean sections of 17.4%. Sweden has a maternal mortality rate of only two per 100,000 live births and a percentage of cesarean sections of 17.3%. Brazil, on the other hand, had a maternal mortality of 260 per 100,000 live births in 2000 and 42.7% of cesarean sections in 2008^15^ ^,^ ^30^.

Some studies have found an inverse association between cesarean rates and maternal and infant mortality in low-income countries, where a significant portion of the population does not have access to basic obstetric care^8^ ^,^ ^25^. In these countries, the provision of appropriate cesarean sections, ensuring better care to the pregnant woman and the newborn, could reduce the chance of complications.

A study conducted in 19 countries evaluating maternal, neonatal, and infant mortality for different percentages of cesarean sections has shown that the curves of neonatal and infant mortality, after adjusting for Gross Domestic Product (GDP) and Human Development Index (HDI), become flat after cesarean rates exceed 10%. Maternal mortality, in turn, appears to increase in cesarean rates above 15%, estimated at 7.8/100,000 for 15% of cesarean sections, 7.9/100,000 for 20%, 8.4%/100,000 for 25%, and 8.8/100,000 for 30%, with an opposite impact to what is often expected^33^.

The option to include only studies that have evaluated cesarean sections without medical indication or women with low obstetric risk makes the results of this review more consistent and reduces the possibility of reverse causality and residual confusion. They are considered the major limitation of studies that have aimed to evaluate the complications associated with cesarean section, since women of higher obstetric risk would be more prone to postpartum complications not necessarily related to the type of delivery.

This result should not be considered dogmatic to define the best practice, but any decision to undergo a major surgery with associated risks should be very well analyzed by all those involved^17^.

This does not exclude the decision-making power of the woman and health professional, as long as the choice is ethical, clarified, and based on reliable evidence, aiming at the best outcome. Health professionals should guide women with clear information, aiming to optimize the well-being of the mother and child binomial and clarify the risks and benefits of each type of delivery in different situations. The choice of the mother, when she initiates this conversation, without the physician offering it, as long as it is enlightened and maintains the safety of the fetus, must be sovereign, respecting her autonomy^20^.

Many women find vaginal delivery risky and a negative experience, while cesarean sections represent better quality care. Over time, women from lower socioeconomic classes also began to adopt behaviors of women of higher classes, taking them as a reference standard and with better quality care, also increasing the cesarean rates in this group^5^. Requests from women to undergo a cesarean section, in the absence of clear biological risks, can often seem irrational; however, previous experiences or reports of traumatic deliveries may justify the choice between a vaginal delivery and a surgical one^5^.

One of the limitations of this review is the inclusion of only observational studies, since there are no randomized clinical trials evaluating the complications associated with the type of delivery in the literature, since it is ethically unacceptable to expose women to supposedly unnecessary cesarean sections. Another limitation is the impossibility of performing a quantitative analysis (meta-analysis) for all the outcomes presented, since few studies are comparable.

This review and meta-analysis may also have been influenced by publication bias, when there is a tendency for published results to be different from reality, as not all the research results are published, either by the decision of the author or financier or editors of scientific journals who may not be interested in publishing negative or non-statistical results. The presence of this bias can be identified by funnel plots and statistical tests, but they are recommended when ten or more studies are included^24^, unlike this review.

Most studies in this review were conducted in high-income countries, and this limits the extrapolation of results to countries and regions with different socioeconomic characteristics.

Future work, especially prospective cohorts of women with low obstetric risk, may have an important impact on the confidence of the effect estimates and greater consistency of results.

We therefore conclude that cesarean sections should be performed with caution. The main challenge related to cesarean sections is its best use, which on the one hand is an important resource for the reduction of maternal and neonatal mortality, but on the other, when used excessively, may be associated with an increased risk of serious maternal outcomes^27^.
